# Evolution of gender representation among Canadian OTL-HNS residents: a 27-year analysis

**DOI:** 10.1186/s40463-017-0232-0

**Published:** 2017-08-29

**Authors:** Sarah Chorfi, Joseph S. Schwartz, Neil Verma, Meredith Young, Lawrence Joseph, Lily H. P. Nguyen

**Affiliations:** 1 Faculty of Medicine, McGill University Health Centre, Montreal Children’s Hospital, McGill University, Room A02.3015, 1001 Boulevard Decarie, Montreal, QC H4A 3J1 Canada; 2 Department of Otolaryngology-Head and Neck Surgery, McGill University, Montreal, Canada; 3 Centre for Medical Education, McGill University, Montreal, Canada; 4 Department of Medicine, McGill University, Montreal, Canada; 5 Department of Epidemiology and Biostatistics, McGill University, Montreal, Canada

**Keywords:** Gender, Female, Diversity, Minority, Otolaryngology, Residents

## Abstract

**Background:**

The proportion of females enrolling into medical schools has been growing steadily. However, the representation of female residents among individual specialties has shown considerable variation. The purpose of this study was to compare the trends of gender representation in Otolaryngology – Head and Neck Surgery (OTL-HNS) residency programs with other specialty training programs in Canada. In order to contextualize these findings, a second phase of analysis examined the success rate of applicants of different genders to OTL-HNS residency programs.

**Method:**

Anonymized data were obtained from the Canadian Residency Matching Service (CaRMS) and from the Canadian Post-M.D. Education Registry (CAPER) from 1988 to 2014. The differences in gender growth rates were compared to other subspecialty programs of varying size. Descriptive analysis was used to examine gender representation among OTL-HNS residents across years, and to compare these trends with other specialties. Bayesian hierarchical models were fit to analyze the growth in program rates in OTL-HNS based on gender.

**Results:**

CaRMS and CAPER data over a 27 year period demonstrated that OTL-HNS has doubled its female representation from 20% to 40% between 1990 and 1994 and 2010-2014. The difference in annual growth rate of female representation versus male representation in OTL-HNS over this time period was 2.7%, which was similar to other large specialty programs and surgical subspecialties. There was parity in success rates of female and male candidates ranking OTL-HNS as their first choice specialty for most years.

**Conclusions:**

Female representation in Canadian OTL-HNS residency programs is steadily increasing over the last 27 years. Large variation in female applicant acceptance rates was observed across Canadian universities, possibly attributable to differences in student body or applicant demographics. Factors influencing female medical student career selection to OTL-HNS require further study to mitigate disparities in gender representation and identify barriers to prospective female OTL-HNS applicants.

## Background

The proportion of female students pursuing postgraduate medical education has grown steadily [1]. Conversely, there has been considerable variation in the proportion of female residents across individual specialties. Studies have found that gender has an influential role in future career and residency program choices, more so than life goals, career motivation and personality traits [1–7].

There are several advantages of gender equity among the physician workforce, including benefits to patient care. Specifically, a diverse workforce includes a broader range of physicians which increases the likelihood of addressing health inequalities and providing care to underserved populations [8]. Although the factors leading to patient satisfaction are complex, male and female physicians tend to adopt different styles of practice, which may be unique and beneficial to patient populations [9]. The benefits of having a gender balanced healthcare team include providing a mixture of complementary interpersonal skills [9]. Research has demonstrated female leaders tend to adopt a more democratic and participative style of leadership [10], a style that is increasingly promoted as the preferred style of leadership given the shift of medicine to a system-based delivery of care. Several studies have reported that female physicians are more likely to engage in a patient-centered approach and spend a greater length of time with their patients [11, 12]. While this is not a gender-specific trait, a gender balanced medical team could thus be more likely to address the needs of a broader demographic of patients. Ultimately, gender parity within the physician workforce encourages the development of a healthcare environment that will strengthen physician-patient relationships, patient satisfaction and overall patient outcomes [13–15].

Gender equity appears to be of considerable benefit beyond obvious moral and ethical interests. Gender misrepresentation may place limitations on the quality of selected resident trainees by inherently limiting the application process to a segregated pool of applicants [5]. A specialty that is attractive to both genders would therefore present more opportunities to select the most qualified candidate. In addition, female role-models for female trainees could encourage pursuit of careers in a broad range of specialties [3, 9].

When specifically considering Otolaryngology - Head and Neck Surgery (OTL-HNS), women were underrepresented compared to larger programs despite increasing female representation in OTL-HNS within American residency training programs [16, 17]. Investigating the demographic characteristics of newly admitted residents allows for a projection of future gender representation across a variety of medical and surgical specialties, helps contextualize physician human resource planning, but also helps identify some potential barriers to increasing gender representativeness within OTL-HNS programs.

To our knowledge, there has been no published study on the nature or evolution of gender representation among Canadian Otolaryngology-Head and Neck Surgery (OTL-HNS) residents. The overarching goal of this study was to provide a descriptive analysis of the recent history of gender representation in residency training in Canada compared to OTL-HNS. We chose to compare Canadian OTL-HNS residency training programs to programs of similar and larger number of registered residents with a focus on growth rates of female trainees compared to male trainees. A secondary aim involved a comparison of acceptance rates to OTL-HNS residency training programs between male and female applicants across all Canadian institutions.

## Methods

Ethics approval was not required since this study was a post-hoc analysis of publically available data. Each postgraduate Canadian OTL-HNS residency program (*n* = 13) was assigned a randomly generated number between one and 13 in order to anonymize institutions for the purposes of analysis.

### Study overview

We conducted a retrospective review of two databases: the Canadian Residency Matching Service (CaRMS) and the Canadian Post-M.D. Education Registry (CAPER). The Canadian Residency Matching Service (CaRMS) provided the following data from 2006 to 2014: gender representation of Canadian residency training programs, demographics of all Canadian OTL-HNS residency training programs, and demographics of medical students applying to Canadian OTL-HNS residency training programs. The CaRMS database captures all applicants participating in the annual CaRMS residency matching process. A matched candidate refers to a candidate who is accepted into a residency program to which they applied.

CaRMS data prior to 2006 was deemed unreliable due to an incomplete data set. Therefore, the Canadian Post-M.D. Education Registry (CAPER) provided Canadian resident demographics including gender representation from 1988 to 2005. This data stems from a self-reported census completed by all resident trainees and submitted by the postgraduate medical education offices of each Canadian medical school to CAPER for archiving.

We measured female representation by analyzing self-reported gender for CAPER data and using the statistics provided by CaRMS. Trends in female representation among Canadian OTL-HNS residency programs were compared to other similarly-sized surgical subspecialties, such as cardiac surgery, neurosurgery, ophthalmology, orthopedic surgery, plastic surgery and urology. Comparisons were also made to larger training programs such as family medicine, internal medicine, general surgery, anaesthesiology, pediatrics and psychiatry. Difference in growth rates between genders in OTL-HNS programs was calculated and compared to both similarly-sized surgical subspecialties as well as larger-sized residency programs. Finally, we compared success rates of female and male applicants and analyzed differences across institutions.

#### Statistical analysis

Descriptive statistics and graphical trends over time were compiled for each residency training program across all years. Bayesian hierarchical Poisson regression models were fit in order to analyze trends over time and compare these trends between males and females across different residency training programs. At the first level of our hierarchical model, the count for each program within each year and for each gender were assumed to follow a Poisson distribution with a rate lambda, assumed different for each data point. Poisson rates were therefore permitted to vary for each year, program and gender. At the second level of our hierarchical model the natural logarithm of these rates followed a linear regression model, with each rate lambda regressed based on program, year, and gender. In order to account for different baseline levels within each program, different intercepts were used in the linear regression model for each program. Similarly, to account for different trends over time within each specialty, each were also given their own slope. At the third level of the hierarchical model non-informative prior densities were used for all parameters, so that the data drive the final inferences. To interpret the estimated regression coefficients on the count scale rather than on the logarithms of these counts, the exponential of the estimated coefficients were calculated. All parameters were estimated using the posterior median with 95% credible intervals. Credible intervals are the Bayeisan analogue to frequentist confidence intervals, but have the more natural interpretation that the probability of the estimated parameter is within the given interval is 95%. All analyses were carried out using WinBUGS (Version 1.4.3, MRC Biostatistics Unit, Cambridge UK).

## Results

### Growth rates of gender representation: OTL-HNS compared to larger programs

**Fig. 1:**
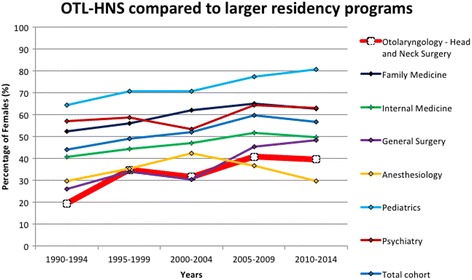
Female representation in OTL-HNS vs larger residency programs. Between 1990 and 2014, the proportion of female residents was averaged over four-year intervals for OTL-HNS and larger residency programs. The averaged data was plotted as a function of time from 1990 to 2014

The proportion of females registered in a residency training program in Canada has increased within all programs included in our analysis, with female representation in OTL-HNS effectively doubling from 20% (i.e. 8 successful female applicants out of a total of 41) to 40% (51 successful female applicants out of a total of 129) between 1990 and 1994 and 2010-2014 (Fig. 1). OTL-HNS had the lowest percentage of females (20%) between 1990 and 1994 compared to larger specialties. Pediatrics had the largest percentage of females (65%) between 1990 and 1994. To account for the increase in size of residency training programs, we analyzed the difference in female and male growth rates of all programs. The difference in growth rates between females and males in OTL-HNS was 2.7% [95% credible interval 0.9, 4.7] per year and was comparable to larger programs (Table 1). In contrast, general surgery and pediatrics had a substantial mean difference in growth rates between females and males (respectively 4.2% [3.1, 5.2] and 3.8% [2.7, 5.0]) (Table 2), which was not significantly different compared to OTL-HNS. Overall, OTL-HNS has a similar difference in female and male growth rates to larger residency training programs and programs of comparable size.

**Table 1:** Gender-specific growth rates in OTL-HNS versus larger residency programs

Specialty	Annual growth rates (percentage/year) [95% credible intervals]		
	Female	Male	Difference
OTL-HNS	8.2 [6.5, 10.1]	5.5 [4.1, 6.9]	2.7 [0.9, 4.7]
Anesthesiology	6.8 [5.8, 7.7]	4.8 [4.0, 5.5]	2.0 [0.8, 3.2]
Family Medicine	4.5 [4.2, 4.7]	2.1 [1.8, 2.4]	2.4 [2.0, 2.7]
General Surgery	3.5 [2.7, 4.3]	−0.68 [−1.3, −0.1]	4.19 [3.1, 5.2]
Internal Medicine	5.0 [4.6, 5.5]	2.5 [2.1, 2.9]	2.5 [1.9, 3.1]
Pediatrics	5.6 [4.9, 6.2]	1.8 [0.8, 2.7]	3.8 [2.7 5.0]
Psychiatry	6.2 [5.6, 6.9]	4.7 [3.9, 5.4]	1.5 [0.5, 2.5]

Data collected from both CaRMS and CAPER regarding the number of female and male applicants in OTL-HNS and selected larger residency programs was averaged between 1990 and 2014. Gender-specific differences in growth rate were calculated for each specific program

**Table 2:** Gender-specific growth rates in OTL-HNS versus surgical subspecialty programs

Specialty	Annual growth rates (percentage/year) [95% credible intervals]		
	Female	Male	Difference
OTL-HNS	8.2 [6.5, 10.1]	5.5 [4.1, 6.9]	2.7 [0.9, 4.7]
Cardiac surgery	5.1 [1.4, 9.2]	2.4 [−0.88, 5.7]	2.7 [0.3, 5.7]
Neurosurgery	6.0 [3.4, 8.5]	3.9 [2.2, 5.6]	2.2 [−0.5, 4.1]
Ophthalmology	6.9 [5.3, 8.5]	4.5 [3.2, 5.7]	2.5 [0.6, 4.2]
Orthopedics	9.2 [7.5, 10.9]	6.4 [5.5, 7.3]	2.8 [1.1, 4.6]
Plastic surgery	8.8 [6.4, 11.6]	5.1 [3, 7.1]	3.6 [1.5, 7]
Urology	8.0 [5.8, 10.2]	5.7 [4.3, 7.1]	2.4 [0.1, 4.4]

Data collected from both CaRMS and CAPER regarding the number of female and male applicants in OTL-HNS and selected surgical specialty programs was averaged between 1990 and 2014. Gender-specific differences in growth rate were calculated for each specific program

### Growth rates of gender representation: OTL-HNS compared to surgical specialties

**Fig. 2:**
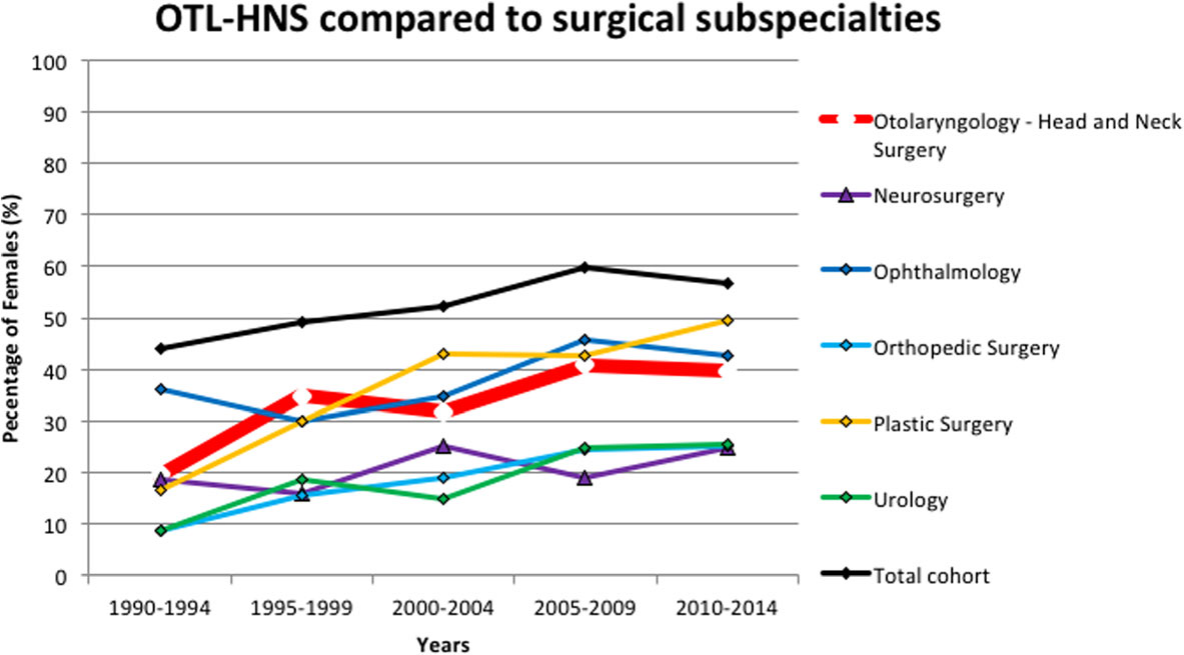
Female representation in OTL-HNS vs surgical subpecialty programs. Between 1990 and 2014, the proportion of female residents was averaged over 4 year intervals. This process was applied to OTL-HNS and surgical subspecialty programs of similar size. The averaged data was plotted as a function of time from 1990 to 2014

The annual growth rate of female representation in OTL-HNS was statistically comparable to most other surgical subspecialties (Fig. 2). There was no statistical difference in annual growth rates of female representation between OTL-HNS compared to cardiac surgery, neurosurgery, ophthalmology, plastic surgery or orthopedic surgery (Table 2). No statistically significant difference was found between female and male growth rates between different surgical subspecialties (Table 2).

### Acceptance rates of first-choice applicants to OTL-HNS

**Fig. 3:**
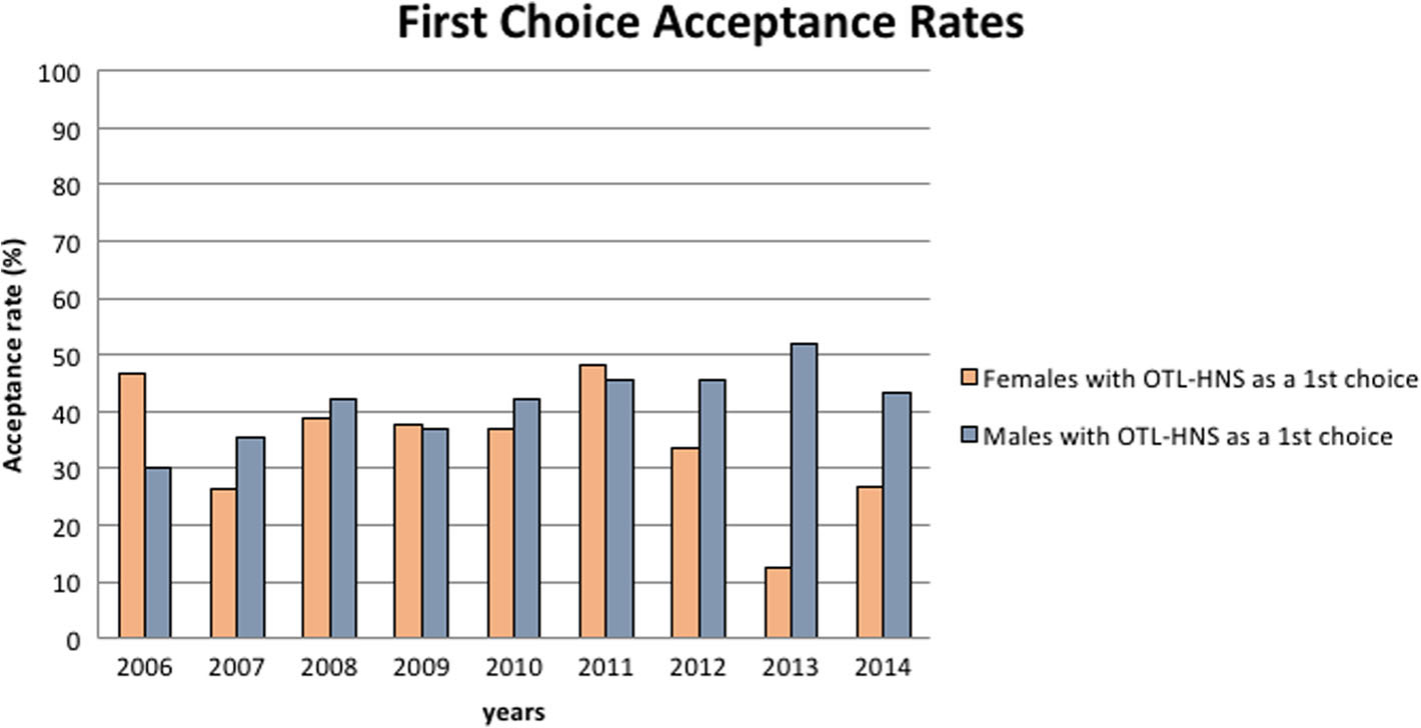
Acceptance rates among first choice applicants to Canadian OTL-HNS programs. Between 2006 and 2014, the proportion of either males or females who ranked OTL-HNS as their first-choice and were accepted into postgraduate residency programs was tabulated. These results were computed according to gender and gender-specific acceptance rates were plotted over this period of time

**Fig. 4:**
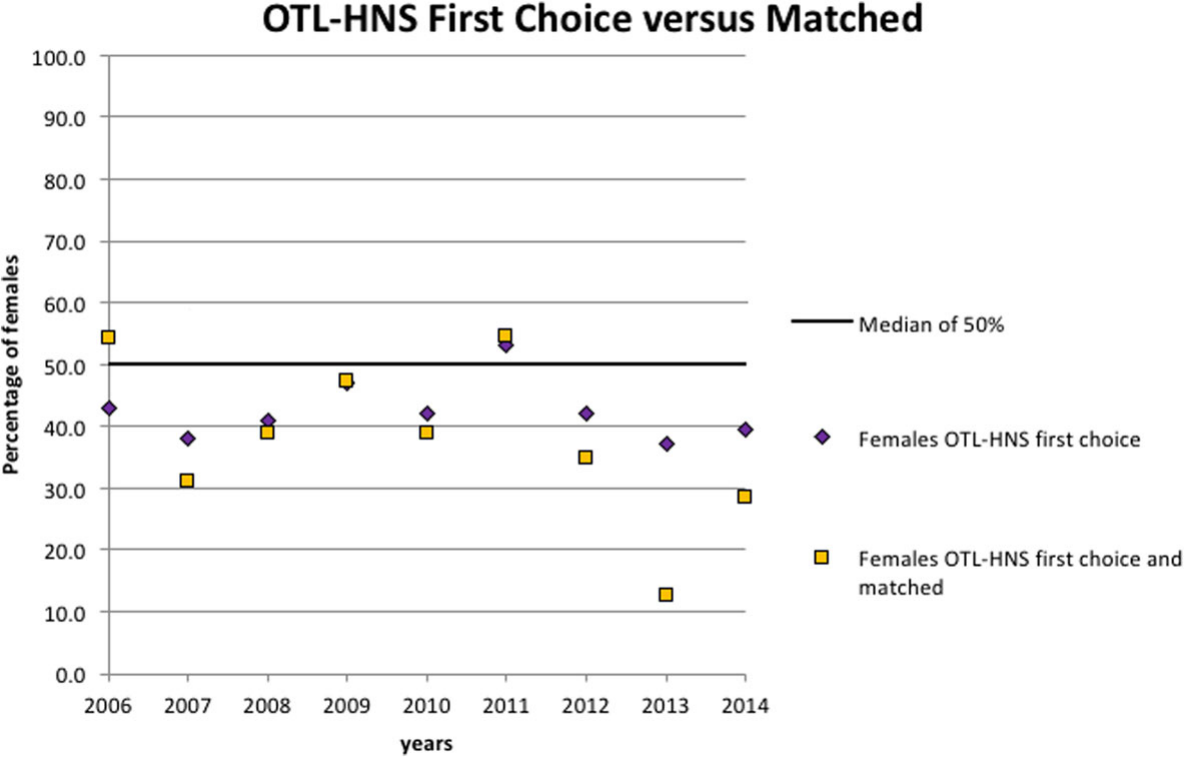
Trends of female representation and acceptance rates in OTL-HNS. Between 2006 and 2014, the proportion of females among all applicants that ranked OTL-HNS as their first choice was calculated and plotted. The proportion of female applicants to OTL-HNS that successfully matched to OTL-HNS postgraduate residency programs was also plotted between 2006 and 2014. The average of all acceptance rates between 2006 and 2014 was also plotted

Acceptance rates among first choice applicants varied among female applicants from year to year, with a decrease compared to male applicant acceptance rates in 2013 and 2014 (Fig. 3). During this period, 12.5% and 28.6% of women ranking OTL-HNS as their first choice were accepted compared to 51.8% and 43.5% of men, respectively (Fig. 4). There was an average of 19 female first-choice applications per year compared to 26 male first-choice applications between 2006 and 2014 (Table 3). The range of acceptance rates for men was 30% to 51.8% and the range for women was 12.5% to 48%. In the 9 years studied, six of these years had a greater acceptance rate of male applicants, and 3 years had a greater acceptance rate of female applicants. Of note, in 2006, 46.6% of females ranking OTL-HNS as their first choice (7 successful candidates out of 15 total candidates) were accepted compared to 30% of males (6 successful candidates out of 20 total candidates). However, no significant differences were found between the acceptance rates of females and males throughout the years studied.

**Table 3:** Acceptance rates among first choice applicants to OTL-HNS programs

Year	Applicant 1st Choice		Matched 1st Choice		Success Rate Female (%)	Success Rate Males (%)
	Female	Male	Female	Male		
2006	15	20	7	6	46.7	30.0
2007	19	31	5	11	26.3	35.5
2008	18	26	7	11	38.9	42.3
2009	24	27	9	10	37.5	37.0
2010	19	26	7	11	36.8	42.3
2011	25	22	12	10	48.0	45.5
2012	24	33	8	15	33.3	45.5
2013	16	27	2	14	12.5	51.9
2014	15	23	4	10	26.7	43.5
Average	19.4	26.1	6.8	10.9	34.1	41.5

Between 2006 and 2014, the proportion of either males or females who ranked OTL-HNS as their first-choice and were accepted into postgraduate residency programs was tabulated. Gender-specific success rates were calculated for each year along with an average success rate

### Female representation and acceptance rates of applicants to OTL-HNS programs

**Fig. 5:**
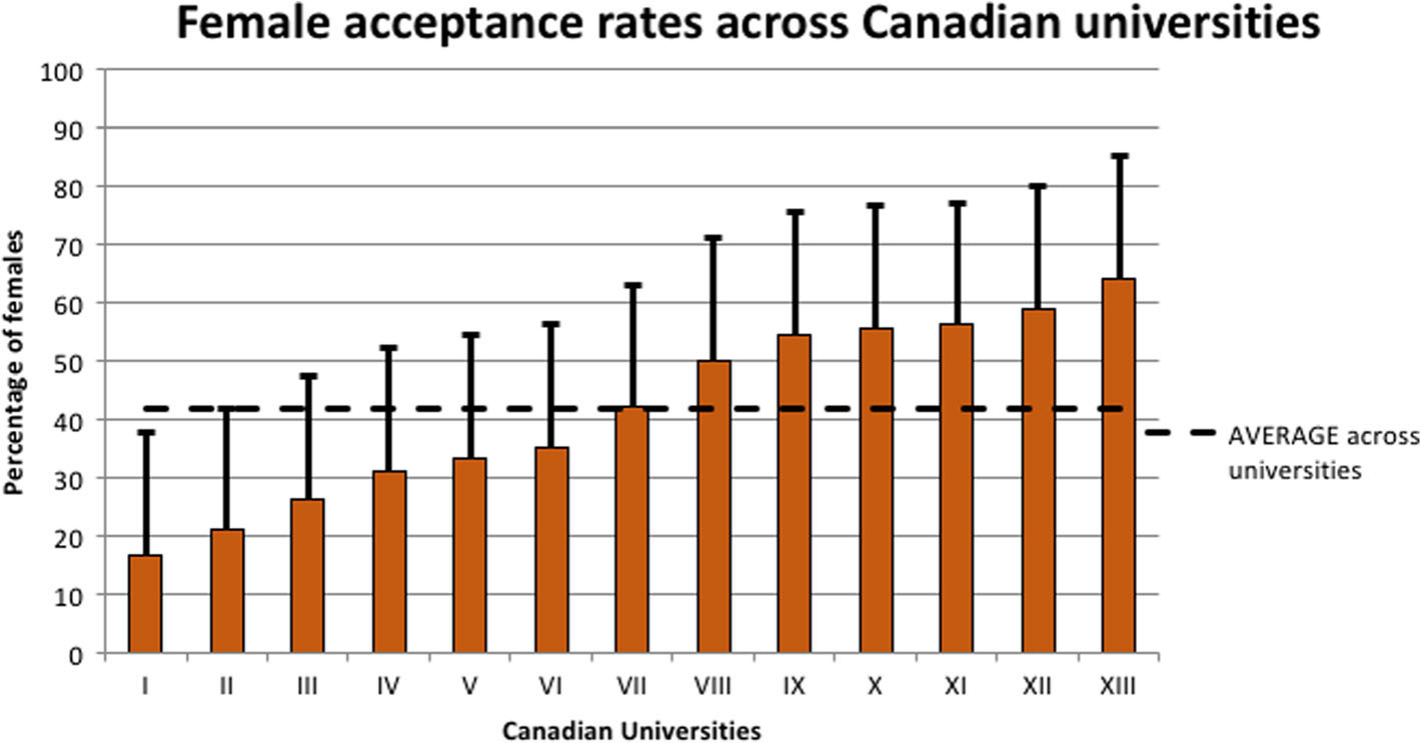
Female acceptance rates into OTL-HNS Canadian universities. Between 2006 and 2014, the proportion of females among all applicants to OTL-HNS that were accepted into postgraduate residency programs was collected for 13 individual Canadian universities. Data from universities were anonymized and plotted in increasing order

Between 2006 and 2014, acceptance rates of female applicants to OTL-HNS varied significantly between universities with a range of 16% to 65%. (Fig. 5). Female representation across institutions was largely above 40% with three programs having less than 30% of their residents being female. One institution had female residents accouting for only 18% of all trainees. However, six of the 13 surveyed institutions had greater than 50% of their residents being female.

## Discussion

To our knowledge, our study is the first to examine the current state and evolution of gender representation among Canadian OTL-HNS training programs in comparison to other postgraduate training programs. Our results document that, currently, OTL-HNS continues to have a lower female resident representation compared to larger training programs, while having similar gender representation in comparison to similarly-sized surgical subspecialties. Interestingly, annual growth rates for female OTL-HNS residents significantly exceeds the rates seen in larger residency programs and the majority of surgical subspecialties. Finally, there exists significant variability in female representation amongst the individual Canadian OTL-HNS residency programs.

Research has explored the role of gender in medical students’ choice of specialty [1–7], with the role model hypothesis suggests that the establishment of gender congruent role models may influence a medical trainee’s choice of specialty. A lack of female role models in certain specialties likely accounts for fewer females in those fields [3, 9], and the impact of resident role models exceeds that of faculty role models, possibly due to greater frequency of interactions and a greater sense of identification with residents than with faculty members [18]. However, it is conceivable that a female medical student from a gender balanced OTL-HNS faculty might have been accepted to a program with a paucity of female resident trainees. The impact of gender congruent role models would require an analysis of the gender makeup of faculty encountered during applicants’ medical school training and current residency program, but is a factor worthy of consideration. This represents an important additional avenue for future investigation to ultimately better understand trends gender representation and to develop gender parity in our healthcare workforce.

In addition, surgical specialties have been rated poorly with respect to work-life balance and this has been traditionally suggested to explain the lack of female representation in surgical specialties [19, 20]. The perception of worse lifestyle in surgery can be associated with the number of working hours, training years prior to certification as well as the acute, stressful nature of the work involved [21, 22]. However, it is unclear how much influence the perception of a poorer lifestyle has on deterring prospective applicants or if this influence is gender-specific [9]. Assumptions regarding lifestyle preferences and gender may not be universally supported, as obstetrics and gynecology attracts a greater number of female applicants despite being known for a less than favorable lifestyle [23].

The reinforcement of gender roles expressed through subtle and unconscious gender beliefs may also influence an applicant’s choice of specialty - male medical students may be more likely to be advised to prioritize specialty preference over familial consideration compared to females [24]. Selection of career choices may also be related to the evaluations of medical students, which may be gender-specific. For example, the student evaluations tend to emphasize particular qualities in trainees that were gender-specific; males have been shown to receive evaluations which reinforce their technical abilities, whereas female candidates receive evaluations which highlight their humanistic attributes [25]. The importance of technical abilities for a career in surgery may disadvantage and discourage applicants who do not receive positive feedback regarding those skills from applying to surgical post-graduate training programs.

The variation in female representation in OTL-HNS residents across Canadian institutions is large and may be attributable to certain factors particular to institutions. With the majority of institutions reaching near parity in female and male representation, the presence of significant underrepresentation in certain institutions suggest that their individual qualities may be more contributory than the characteristics of OTL-HNS as a specialty. Female representation in undergraduate medical education programs and the lack of postgraduate role models across the country may help explain differences in gender representation at the postgraduate level, however this remains speculative at best. The differences in gender composition of the student body may also be in due to differences in recruitment and admissions policies, and remains an important avenue for future research. Further studies may be of benefit to post-graduate OTL-HNS program directors in so far as facilitating gender parity among recruited trainees, including the identification of barriers at the application and admissions stages of post graduate medical education.

Strengths of our study include analysis over a prolonged time frame to allow assessment of growth of female representation. Contextualization of gender representation in OTL-HNS was possible through comparison with larger programs and smaller surgical subspecialty programs, which share a number of similar characteristics with OTL-HNS. The main limitation of this study was the assessment of gender representation trends in smaller programs, as there are very few admitted candidates per year. OTL-HNS and other surgical subspecialty programs have a relatively small number of trainees, such that minor changes in the number of female trainees may translate to a large change in the rate of female representation across time. As a result, it is difficult to determine the significance of increased growth rates in female representation in OTL-HNS residency, particularly when comparisons are made to larger programs. Small changes in gender composition of the trainees may lead to large variation in measured female representation and growth rates of female applicants, however our results demonstrated relative consistency across time. The incorporation of two separate sources of data (CAPER and CaRMS) for our analysis with CAPER being a self-reported census represents an additional limitation of this study.

Future work may include investigating the influence of medical curriculum and role model exposure on trainees’ selection of career paths during pre-clinical years. The investigations of other contributory factors such as gender role models, applicant perception of the given specialty, perception of lifestyle within the given specialty and the recruitment techniques employed by residency programs also represent interesting avenues of future study, and could provide explanations for the findings described here.

## Conclusion

Female representation in OTL-HNS continues to improve with a large variability in the success rate of female applicants across Canadian institutions. Despite progress towards gender parity in OTL-HNS residency programs in Canada, certain institutions continue to report underrepresentation of female residents, possibly due to certain characteristics unique to those residency programs. A better understanding of these trends may allow us to identify supports and barriers of gender parity in surgical training programs and ultimately suggest appropriate recommendations. This will lead to the most qualified trainees being selected, gender parity within the OTL-HNS workforce, and ultimately improved patient outcomes. Ensuring the presence of gender congruent role models and addressing the negative perception of work-life balance within subspecialty surgery could reduce barriers to female medical student trainees applying to OTL-HNS residency training programs, and careful monitoring or admissions practices may facilitate the matching of the most appropriate candidates, regardless of gender.

## Abbreviations

CAPER: Canadian Post-M.D. Education Registry
CaRMS: Canadian Residency Matching Service
OTL-HNS: Otolaryngology – Head and Neck Surgery
